# Wearable Device for Cumulative Chlorobenzene Detection and Accessible Mitigation Strategies

**DOI:** 10.3390/s23187904

**Published:** 2023-09-15

**Authors:** Aryan Mago, Yeon-Suk Yang, Jae-Hyuck Shim, Aijaz Ahmad John

**Affiliations:** 1 Department of Medicine, Division of Rheumatology, University of Massachusetts Chan Medical School, Worcester, MA 01655, USA; 2 Horae Gene Therapy Center, University of Massachusetts Chan Medical School, Worcester, MA 01655, USA; 3 Li Weibo Institute for Rare Diseases Research, University of Massachusetts Chan Medical School, Worcester, MA 01655, USA

**Keywords:** chlorobenzene, chronic exposure, osteopenia, volatile organic compounds (VOCs), wearable sensor, machine learning (ML), vitamin D, smartphone integration

## Abstract

Chronic exposure to low concentrations of volatile organic compounds (VOCs), such as chlorobenzene, is not being monitored in industrializing countries, although VOC exposure is associated with carcinogenic, organ-toxic, and endocrine-disrupting effects. Current VOC-sensing technologies are inaccessible due to high cost, size, and maintenance or are ineffective due to poor sensitivity or reliability. In particular, marginalized individuals face barriers to traditional prescription VOC treatments due to cost, lack of transportation, and limited access to physicians; thus, alternative treatments are needed. Here, we created a novel cumulative wearable color-changing VOC sensor with a paper-based polydiacetylene sensor array for chlorobenzene. With a single smartphone picture, the sensor displays 14 days of logged chlorobenzene exposure data, interpreted by machine-learning (ML) techniques, including principal component analysis. Further, we explored the efficacy of affordable and accessible treatment options to mitigate a VOC’s toxic effects. Vitamin D and sulforaphane are naturally found in cruciferous vegetables, like broccoli, and can be used to treat chlorobenzene-mediated bone degradation. Our platform combines these components into a smartphone app that photographs the sensor’s colorimetric data, analyzes the data via ML techniques, and offers accessible treatments based on exposure data.

## 1. Introduction

Volatile organic compounds (VOCs) are a class of chemicals characterized by their high vapor pressure and low boiling point [1]. VOCs are used as major components of various household and commercial products, including paints, dyes, adhesives, cleaners, and plastics, as well as petroleum products, such as gasoline. VOCs have been suggested to pose long-term human health risks, including carcinogenic, organ-toxic, and endocrine-disrupting effects [2,3,4]. Recently, the detrimental health effects of VOCs have been addressed by new regulations in developing countries. Countries such as India and Bangladesh have health legislation to limit VOC exposure [5]. Accumulating evidence has demonstrated that certain individuals have a higher risk of exposure to VOCs than is advisable by national guidelines and standards, for example, in certain occupations, such as painting, printing, and manufacturing [6,7]. In addition, ambient air pollution kills 4.2 million individuals per year, and 89% of these premature deaths occur in low-to-middle-income countries (LMICs) [8]. There are significant barriers that prevent workers from seeking care for VOC exposure, including lack of awareness, transportation, affordability, time, and medication availability [9,10,11,12]. Although VOCs are a major health issue worldwide for LMIC populations, people are often unaware of their exposure to VOCs, and those who are aware often lack access to healthcare to receive treatment.

Current VOC detection methods include gas chromatography–mass spectrometry (GC-MS), photoionization detection (PID), flame ionization detection (FID), and metal oxide semiconductors (MOSs) [13]. However, the use of these methods is limited due to a combination of cost, size, weight, sensitivity, maintenance, reliability, or a lack of cumulative or personal detection [14,15]. These limitations make current VOC detection technologies impractical or ineffective, particularly in remote or resource-sparse regions [16].

Various advanced materials technologies have recently been explored for chemical detection, such as Surface Plasmon Resonance, including graphene, metamaterial absorbers, and nanoring cavity sensors [17,18,19,20]. Polydiacetylenes (PDAs) have recently been explored as an alternative VOC detection method because of their color-changing response upon exposure to aqueous and gaseous VOCs via a mechanism known as solvatochromism [21]. Upon exposure to various environmental stimuli, PDAs change color from blue to shades of red. PDAs consisting of diacetylene (DA) monomers that vary in length, functional groups, and polarity produce different colorimetric responses (e.g., shades of pink and orange) to a certain stimulus. Solutions saturated with DA monomers can be drop-casted or inkjet-printed onto paper, thin films, or 3D scaffolds [22]. The structure is then polymerized into an active PDA via UV exposure. Therefore, arrays of different PDAs have been combined to produce a unique colorimetric response—a “fingerprint”—for each VOC exposure [21]. The formation of PDA arrays is highly reproducible and their performance is highly consistent, as illustrated by a variety of studies [21,22,23,24,25,26].

A previous study developed an array of PDAs that identify 18 different VOCs with 100% accuracy using principal component analysis (PCA) [27]. In addition, a smartphone app has been developed that recognizes 11 different organic solvents from the colorimetric response of a four-dot PDA array by comparing the redness and hue values of each dot to a preloaded database [26]. However, there are multiple limitations to these systems. For example, PDAs have not been used in this context to detect the concentration or time of exposure to VOCs [22]. Additionally, airborne emissions are typically described through a time-weighted average, which combines the two factors [28], and a detection system for cumulative amounts of VOC exposure has not been tested [22]. Moreover, the detection system has not been mobilized into a wearable apparatus [22,24]. Finally, these apps do not provide accessible treatment options for VOC exposure. Barriers, such as cost, insurance, transportation, and access to a doctor, may prevent LMIC individuals from accessing prescription medication as treatment. Developing a diet or over-the-counter (OTC) supplement-based treatment would provide VOC treatment options to individuals without access to a pharmacy or doctors.

In this study, we developed a low-cost, wearable, visual VOC detection apparatus composed of a transparent plastic button pin containing printer paper and chlorobenzene as the target VOC. The paper is dotted with identical arrays of PDAs. After multiple days, the pin can be photographed by a smartphone, and machine-learning techniques can be used to estimate the quantity of the VOC that the individual was exposed to as a time–concentration product. With a preloaded database, the smartphone app can then advise on affordable OTC treatments. The results demonstrate that the system can (1) log multiple days of personal VOC exposure, (2) identify the quantity of the VOC that the individual was exposed to as a time–concentration product, and (3) advise on accessible treatments for the health effects based on the amount of VOC exposure.

## 2. Methods

### 2.1. Choosing DA Monomers

DA monomers were purchased as powders. DA 1 (10,12-pentacosadiynoic acid (PCDA)) was purchased from Sigma-Aldrich (St. Louis, MO, USA), ≥97.0% (HPLC). DA 2 (10,12-tricosadiynoic acid (TCDA)) was purchased from Sigma-Aldrich, ≥98.0% (GC). DA 3 (5,7-Dodecadiynedioic acid) was purchased from Sigma-Aldrich, ≥99.0% (KF). DA 4 (10,12-Docosadiynedioic acid) was purchased from Thermo Scientific Chemicals (Bengaluru, India), ≥95.0% (GC). Each DA powder was added and dissolved in 2-propanol at a concentration of 10 µg/mL. A four-dot array was added to the printer paper, each with 1 µL from its respective solution. Arrays were polymerized with UV light for 1 min each with a UV lamp (254 nm, 500 µW/cm^2^) [27].

### 2.2. Exposure Trials

**Figure 1:**
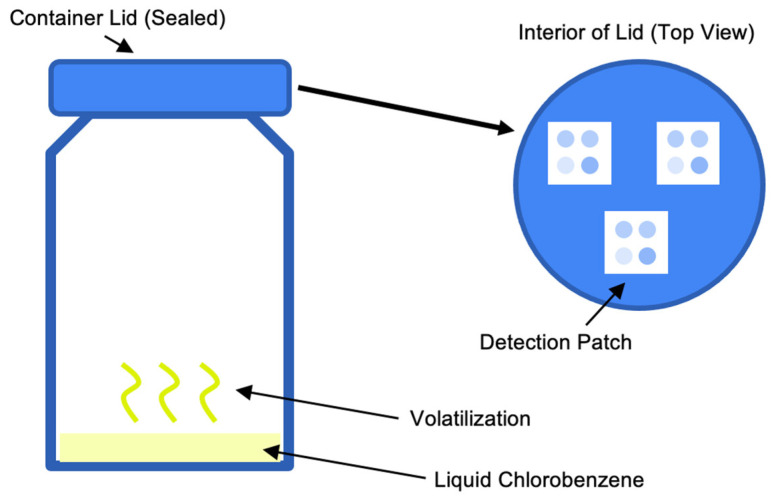
Schematic of VOC exposure apparatus.

To generate training data for the PCA model, three gaseous concentrations of chlorobenzene were used: 10, 20, and 40 ppm. Each group contained three arrays as replicates. Chlorobenzene was deposited as a liquid onto the base of a 100 mL container. The patch was placed on the inside of the cap, and the container was sealed (Figure 1). A closed, sealed system was used to isolate the impact of chlorobenzene. However, pressure, vacuum, and airflow have negligible impacts on the PDA color change [23]. Furthermore, the selectivity of the chosen PDAs to chlorobenzene has been confirmed in prior studies, such as in the presence of gasoline fumes and other VOCs [26,27]. Therefore, the environment may simulate user exposure. The liquid was observed to be completely evaporated within 1 h. Arrays in each group were exposed for 8 h per day for 14 days. Arrays were initially photographed and then photographed daily after exposure under identical lighting conditions.

### 2.3. Colorimetric Response and Analysis

The patches were photographed with a smartphone (iPhone 8) camera. Hue and saturation were adjusted using ImageJ software 1.52k until the background paper was perfectly white. The mean Red–Green–Blue (RGB) values for each of the four PDA dots after VOC exposure were determined, creating a 12-dimensional vector. The RGB values of the dots before VOC exposure were preloaded in the software. Post-exposure RGB values were subtracted from pre-exposure RGB values to create a 12-dimensional ΔRGB vector. Principal component analysis was used to reduce the dimensionality of the data to two principal components (PCs). VOC exposure can be measured as a time–concentration product [29,30]. So, the gaseous concentration (ppm) was multiplied by hours of exposure (h) to acquire an exposure quantity in ppm-hours. The quantity of exposure was graphed against the distance along the first principal component, and a regression line was fit. Each daily photograph in each of the three replicates at each of the three concentrations was used as training data. An additional replicate was used to test the accuracy of the method.

### 2.4. Effects of Chlorobenzene Exposure on Osteoclast Development

For the osteoclast differentiation assay, mouse bone marrow macrophages (BMMs) were harvested from bone marrow cells in the long bones of 8-week-old wild-type mice (C57BL/6J, The Jackson Laboratory, Sacramento, CA, USA). In brief, bone marrow cells were flushed out from the femurs and tibias, treated with red blood cell lysis buffer, and suspended in 10% fetal calf serum (FCS) and 1% penicillin/streptomycin (Corning, New York, NY, USA). Cells were cultured in the presence of M-CSF (10 ng/mL, R&D Systems, Minneapolis, MN, USA, 416-ML), and 1 day later, non-adherent cells were plated at a density of 0.5 × 10^6^ cells/well in 24-well plates. BMMs were differentiated into osteoclasts by treating them with M-CSF (10 ng/mL) and RANKL (20 ng/mL, R&D Systems, 462-TEC), and two days later, liquid chlorobenzene was added daily at four concentrations (0 (control), 1, 10, and 100 µg/mL) for 14 days. To assess osteoclast differentiation, TRAP staining was performed using a leukocyte acid phosphatase staining kit (Sigma, 387A) according to the manufacturer’s protocol. The TRAP-stained osteoclasts were detected using an Evos microscope (Applied Biosystems, Waltham, MA, USA).

### 2.5. Calvarial Explant and Chlorobenzene Exposure

Calvariae were isolated from 3-day-old pups (C57BL/6J, Jackson Laboratory) and cultured in α-minimal essential medium (αMEM) supplemented with 10% FCS, 1% penicillin/streptomycin (Corning), ascorbic acid (200 μM, Sigma, A8960), and β-glycerophosphate (10 mM, Sigma, G9422). Permutations of chlorobenzene, sulforaphane, Vitamin D, and bisphosphonate alendronate were added daily as the medium was changed. Liquid chlorobenzene was added to wells of the 12-well plate containing the calvariae to achieve a liquid concentration of 10 μg/mL. The concentration of sulforaphane added to the serum was equivalent to the estimated serum concentration during the consumption of three cups of raw glucoraphanin-rich broccoli: 0.5 µM [31]. Alendronate was added in an amount corresponding to the serum concentration of a traditional treatment. Vitamin D was added to the medium with 1.0 × 10^−9^ Molarity (M) for eight days.

### 2.6. Quantitative RT-PCR Analysis

Total RNA was isolated from the calvariae using QIAzol (QIAGEN, Hong Kong, China), followed by quantification via NanoDrop One (Thermo Scientific). Further, cDNA synthesis was carried out using the high-capacity cDNA reverse transcription kit (Applied Biosystems, Waltham, MA, USA). RT-PCR analysis was performed for osteoclastogenic markers, like nuclear factor kappa B (NF-κB), matrix metallopeptidase 9 (Mmp-9), cathepsin K (Ctsk), nuclear factor of activated T-cell cytoplasmic-1 (Nfatc-1), and chemokine ligand 2 (Ccl-2), using SYBR Green PCR master mix (Bio-Rad, Hong Kong, China) with a CFX Connect RT-PCR detection system (Bio-Rad).

### 2.7. Statistical Analysis

The qPCR results were interpreted by one-way ANOVA, followed by Sidak’s Multiple Comparison Test. Values with *p* < 0.05 were considered statistically significant. Graphed data of qPCR results were represented as mean ± one standard deviation (SD).

## 3. Results

### 3.1. Developing an Affordable VOC Detection Patch

To develop an affordable, portable, and accurate VOC detection system, we constructed a VOC patch using different diacetylene (DA) pigments. Four DA monomers were chosen to create the PDA array based on the findings of Eaidkong et al. [27]. Two of the DAs were traditional amphiphilic DAs that vary in length, with a methyl group on one end and a carboxyl group on the other: 10,12-pentacosadiynoic acid (PCDA) and 10,12-tricosadiynoic acid (TCDA) (DAs 1 and 2, respectively). The other two DAs (DAs 3 and 4) were trans-isomers that vary in length: 5,7-Dodecadiynedioic acid and 10,12-Docosadiynedioic acid. DAs 3 and 4 were chosen for their boloamphiphilic nature, meaning that they have hydrophilic carboxyl groups on both ends of the long hydrophobic diacetylene chain [26,27]. In addition to the four DAs, the patch was composed of a button pin, hydrogel, and paper. The total cost of the patch was less than USD 0.50 (Table 1), which is 100-fold less expensive than current VOC detection methods of comparable accuracy [16]. 

### 3.2. Correlating the Color Change from the VOC Detection Patch with the Amount and Time of Chlorobenzene Exposure

**Figure 2:**
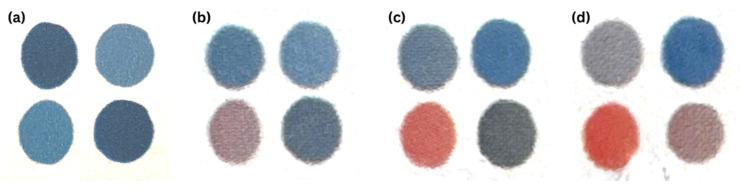
Representative PDA arrays (**a**) before exposure; after (**b**) 7 days of chlorobenzene exposure at 10 ppm, (**c**) 7 days of chlorobenzene exposure at 20 ppm, (**d**) 14 days of chlorobenzene exposure at 20 ppm.

Due to their differing chemical structures, each of the four DAs produced a different colorimetric response profile upon exposure to the VOC chlorobenzene, which could be distinguished by the naked eye (Figure 2). To develop a standardized readout to correlate the color change with the amount of chlorobenzene exposure, we used three different concentrations of chlorobenzene: 10, 20, and 40 ppm. Liquid chlorobenzene was deposited onto the base of a 100 mL container, the VOC detection patch was placed on the inside of the cap, and the container was sealed. The liquid completely evaporated within 1 h. Arrays in each group were exposed for 8 h per day for 14 days. The non-exposed patch was also photographed and used as the background image, and then photographs were taken daily immediately after exposure under identical lighting conditions. No change in the PDA color was observed when unsealing the container. The patches were observed for 7 days after exposure, and no regeneration or color reversion was observed. The reference curve had a coefficient of determination of 0.9544, indicating a strong correlation. The rate of change in PC1 remained constant at each exposure concentration: 1.16/h. at 10 ppm, 2.14/h. at 20 ppm, and 4.31/h. at 40 ppm. The accuracy of the model was tested using data from an additional replicate. Colorimetric analysis of the patch via the smartphone app distinguished chlorobenzene exposure from the control with 100% accuracy. After a single day of exposure, the patch estimated exposure, via an 8 h time-weighted average, within 10% of the true exposure in 92% of trials and within 25% of the true exposure in 100% of trials. With one photograph, the patch could display 14 days of logged cumulative exposure. The patch averaged within 10% of the time–concentration product in 91% of trials and within 25% of the true time–concentration product in 94% of trials (Table 2).

**Figure 3:**
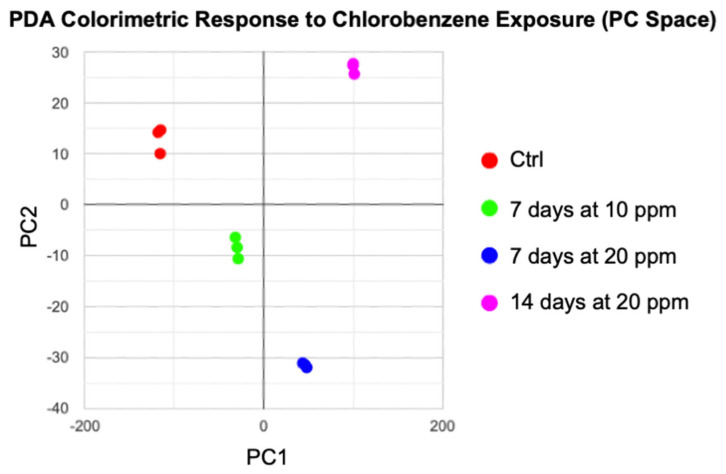
The total colorimetric response of the polydiacetylene (PDA) array to each exposure group of chlorobenzene was reduced and transformed into principal component (PC) space.

**Figure 4:**
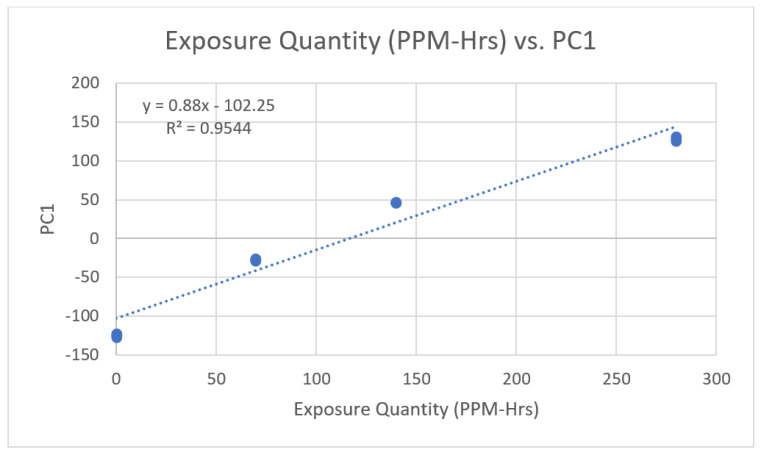
Reference curve for quantity of VOC exposure (in ppm-h) vs. color change pattern of the PDA matrix. The PDA matrix color change is represented by principal component 1 (Figure 3).

To develop a reliable and consistent readout for chlorobenzene detection, we performed a principal component analysis (PCA). In PCA, the first and second principal components (PC1, PC2) accounted for 93.3% and 6.6% of the variance, respectively. Since the distance along principal component (PC) 1 accounts for 93.3% of the variance and is closely correlated with concentration, PC1 (see *x*-axis, Figure 3 and Figure 4) can be used to assess the concentration of chlorobenzene exposure. 

### 3.3. Assessing the Impact of Chlorobenzene Exposure on Osteoclasts

**Figure 5:**
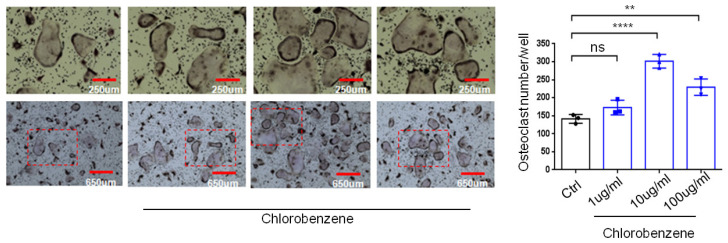
Representative images of osteoclast plates after 7 days of culture, with TRAP staining at 10× and 4× magnification. Osteoclasts were left untreated (control) or exposed to 1 µg/mL, 10 µg/mL, and 100 µg/mL chlorobenzene. The graph indicates the number of osteoclasts per well, as manually counted. All values represent mean ± SD (*n* = 3). Red box: Magnified area. Not significant: ns; *p* < 0.01: **; *p* < 0.0001: ****.

Chlorobenzene exposure may upregulate transcription factors and cytokines that promote bone degradation, such as NF-κB and CCL2 [32,33,34,35]. To determine the impact of chlorobenzene exposure on osteoclasts, the key cell types in bone degradation, we performed a differentiation assay on mouse bone marrow macrophages (BMMs) obtained from bone marrow cells in the long bones of 8-week-old wild-type mice (C57BL/6J). BMMs were differentiated into osteoclasts, and two days later, the BMMs were either untreated or treated with liquid chlorobenzene at four different concentrations: 0 (control), 1, 10, and 100 µg/mL for 14 days. Inhalation of 10 ppm (46 mg/m^3^) of chlorobenzene for 8 h per day over 14 days can cause a blood concentration of 1 ug/mL in humans [36]. We assessed osteoclast differentiation using TRAP staining and determined that chlorobenzene exposure significantly increased osteoclast differentiation in vitro (Figure 5), indicating that chlorobenzene exposure led to an increase in bone degradation.

### 3.4. Assessing the Impact of Different Treatments to Reverse the Effects of Chlorobenzene-Exposed Osteoclasts

**Figure 6:**
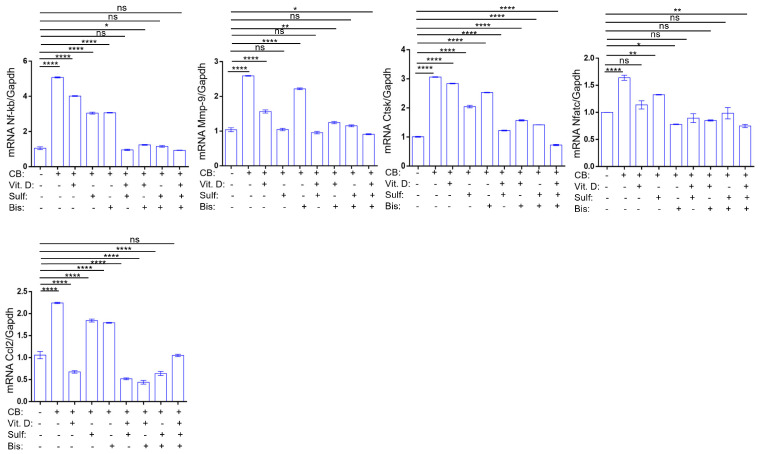
Calvarial explant models were exposed to chlorobenzene and various treatment combinations: sulforaphane, Vit. D, and alendronate. Graphs showing the relative mRNA expression of various osteoclastogenic markers. All values represent mean ± SD (*n* = 3). *p* < 0.05: *; *p* < 0.01: **; *p* < 0.0001: ****.

To identify potential treatment options based on exposure to chlorobenzene, we examined affordable OTC options, including alendronate, Vitamin D, and sulforaphane, on chlorobenzene-exposed osteoclasts. Using a calvarial explant model, we tested these different compounds alone and in combination for their ability to downregulate bone degradation (Figure 6). A combination of bisphosphonate, Vitamin D, and sulforaphane treatment with chlorobenzene was the most effective treatment to mitigate the effects of chlorobenzene exposure on osteoclastogenic markers. This combination decreased the expression of each osteoclastogenic marker to levels equal to or below those of the control. Importantly, we showed that a combination of the OTC compounds sulforaphane and Vitamin D is as effective as the more expensive bisphosphonate treatment.

## 4. Discussion

**Figure 7:**
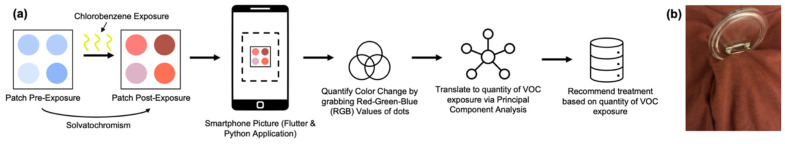
(**a**) Visual model of the working principle and platform of the chlorobenzene detection system, including integration with mobile software; (**b**) patch casing pinned to clothing, demonstrating wearability.

In this study, we demonstrate the feasibility of a PDA-based detection method for VOCs using a patch that produces a distinct color change pattern for chlorobenzene that can be recognized by the naked eye (Figure 7a). The patch costs USD 0.46 to produce, only requiring the pigments, the hydrogel, a button pin, and paper. The materials and the finished product are shelf-stable. The patch’s casing and pin are reusable. The paper and PDA film are biodegradable [22,37]. The device weighs less than 20 g. Due to the casing and pin, the patch is easily wearable (Figure 7b). A single picture of the patch communicates 14 days of logged exposure data, with near-perfect accuracy in the recognition of exposure and high accuracy in determining the concentration of exposure.

The chosen PDAs are highly selective to chlorobenzene, even in the presence of related chemicals, such as other VOCs and gasoline fumes [27]. Future experiments may reduce non-specific interactions by integrating chemicals such as polyethylene glycol with various lengths [27]. Additionally, the patch may be sensitive to low or high humidity and water exposure. A previous study demonstrated a weak colorimetric response from PDA to atmospheric humidity (relative humidity ≈ 80%) at temperatures above 45 °C [25]. Using PCA, the smartphone app can analyze and send the data to a health provider or an Occupational Health and Safety Administrator. 

The detrimental impacts of chlorobenzene exposure have been noted in various countries. For example, garment workers in Bangladesh and Quebec, exposed to dyes commonly containing chlorobenzene, experience significantly lower bone mineral density, greater rates of osteopenia, and greater disability rates than average [38]. In our study, the calvarial explant model confirmed established research that VOCs, such as chlorobenzene, have tangible impacts on normal physiological functions, such as bone degradation. The VOC chlorobenzene is known to upregulate the cytokine CCL-2 and the transcription factor NF-κB in various organs [32,39]. Both CCL-2 and NF-κB are relevant to the formation of osteoclasts, bone-degrading cells.

The existing treatment for osteoporosis is bisphosphonates, such as alendronate, that inhibit osteoclast-mediated bone resorption [40]. Treatment with alendronate effectively mitigated chlorobenzene’s effects. However, these drugs can be expensive or inaccessible for a remote worker. Therefore, we explored a diet and supplementation treatment path to treat chlorobenzene-induced osteoporosis: Vitamin D and sulforaphane, an isothiocyanate naturally found in cruciferous vegetables like broccoli, bok choy, and cabbage [41]. This is consistent with previous studies demonstrating that a combination of Vitamin D and sulforaphane reduced osteoclastogenic activity at a comparable level to alendronate, nearly mitigating the negative health effects of chlorobenzene. OTC (over-the-counter) supplements are sufficient to achieve the serum concentration of Vitamin D given to these calvariae [42,43,44]. In addition, three daily cups of glucoraphanin-rich broccoli in humans can achieve the serum sulforaphane concentration used in the experiment [45]. These results promote research into more accessible treatments for the health effects of VOCs, particularly relevant for remote and socioeconomically marginalized persons.

Natural remedies like diet and supplementation are historically understudied in medical research. However, these treatments provide accessible healthcare to patients with income, transportation, and medical access barriers. This experiment demonstrates the feasibility of our platform in detecting an example VOC and providing treatments to counter its effects. We also support that an OTC remedy is an effective treatment against VOC damage. This encourages similar research for the remaining VOCs. The smartphone app has been programmed to advise on these treatments with or without medication. Further, the advice can be updated for Over-The-Air applications as new research arises.

## 5. Conclusions

Our study combines a unique strategy of detection and mitigation into a smartphone app that photographs the wearable patch, analyzes the colorimetric data, and advises on both prescription and accessible solutions. This VOC detection patch and example experiment illustrate a system that can detect a VOC with minimal cost, effort, and error; analyze and display exposure data for informed diagnoses and personal advocacy; and provide accessible treatment options to overcome medical access barriers. Further research may explore the feasibility of the patch in various conditions, such as humidity and temperature. PDA technology has been applied to detect other VOCs as well. In addition, it is vital to conduct more research into the harms of VOC exposure and the efficacy of OTC treatments. With the newfound support and awareness of the scientific community, this VOC detection system can be advanced to help improve these health issues for a more equitable future.

## Figures and Tables

**Table 1:** Cost breakdown.

Name	Cost Per Patch (USD)
Button pin	USD 0.12
Hydrogel	USD 0.06
DA pigments (1–4)	USD 0.27
Paper	USD 0.01
Total	USD 0.46

**Table 2:** Fraction of trials in which predicted exposure was correct.

	Single Day	Multiple-Day Sum
Accurate within 10% of true exposure	92%	91%
Accurate within 25% of true exposure	100%	94%

## Data Availability

Data supporting the findings of this manuscript are available from the corresponding authors upon request. The raw data are protected and are not available due to data privacy laws. However, the processed generated in this study are provided in the Supplementary File.

## Supplementary Materials

The following supporting information can be downloaded at https://www.mdpi.com/article/10.3390/s23187904/s1, Figure S1: Calculated Eigenvalues of each principal component in the covariance matrix. Eigenvalues signify the portion of the total variance that each Principal Component (each axis in Figure 3) captures; Table S1: Mouse Primer Sequences (RT-PCR) [46,47,48]; Table S2: RGB Color Values of PDA Arrays after Chlorobenzene Exposure, in described groups.

## References

[1] Krishnamurthy A., Adebayo B., Gelles T., Rownaghi A., Rezaei F. (2020). Abatement of gaseous volatile organic compounds: A process perspective. Catal. Today.

[2] Shuai J., Kim S., Ryu H., Park J., Lee C.K., Kim G.-B., Ultra V.U., Yang W. (2018). Health risk assessment of volatile organic compounds exposure near Daegu dyeing industrial complex in South Korea. BMC Public Health.

[3] Eldesouki M.A. (2013). Study of the Effect of Occupational Exposure to Volatile Organic Compounds (VOC’s) on Male Reproductive Hormones. World J. Med. Sci..

[4] National Research Council (2009). Systemic Exposures to Volatile Organic Compounds and Factors Influencing Susceptibility to Their Effects. Contaminated Water Supplies at Camp Lejeune: Assessing Potential Health Effects.

[5] Gaur M., Singh R., Shukla A. (2016). Volatile Organic Compounds in India: Concentration and Sources. J. Civ. Environ. Eng..

[6] Srivastava A., Joseph A.E., Nair S. (2004). Ambient levels of benzene in Mumbai city. Int. J. Environ. Health Res..

[7] Do D.H., Walgraeve C., Amare A.N., Barai K.R., Parao A.E., Demeestere K., van Langenhove H. (2015). Airborne volatile organic compounds in urban and industrial locations in four developing countries. Atmos. Environ..

[8] WHO “Ambient (Outdoor) Air Pollution.” World Health Organization. https://www.who.int/newsroom/fact-sheets/detail/ambient-(outdoor)-air-quality-and-health.

[9] Alford K.L., Kumar N. (2021). Pulmonary Health Effects of Indoor Volatile Organic Compounds—A Meta-Analysis. Int. J. Environ. Res. Public Health.

[10] Byliński H., Gębicki J., Namieśnik J. (2019). Evaluation of Health Hazard Due to Emission of Volatile Organic Compounds from Various Processing Units of Wastewater Treatment Plant. Int. J. Environ. Res. Public Health.

[11] Steinemann A. (2015). Volatile emissions from common consumer products. Air Qual. Atmos. Health.

[12] Syed S.T., Gerber B.S., Sharp L.K. (2013). Traveling Towards Disease: Transportation Barriers to Health Care Access. J. Community Health.

[13] Khatib M., Haick H. (2022). Sensors for Volatile Organic Compounds. ACS Nano.

[14] Chen Y., Du L., Tian Y., Zhu P., Liu S., Liang D., Liu Y., Wang M., Chen W., Wu C. (2022). Progress in the Development of Detection Strategies Based on Olfactory and Gustatory Biomimetic Biosensors. Biosensors.

[15] Liu T., Ding L., Fang Y. (2022). Real-time and wireless monitoring platforms for vital chemicals toward wearable applications. Matter.

[16] Pathak A.K., Swargiary K., Kongsawang N., Jitpratak P., Ajchareeyasoontorn N., Udomkittivorakul J., Viphavakit C. (2023). Recent Advances in Sensing Materials Targeting Clinical Volatile Organic Compound (VOC) Biomarkers: A Review. Biosensors.

[17] Tang B., Li Z., Palacios E., Liu Z., Butun S., Aydin K. (2017). Chiral-Selective Plasmonic Metasurface Absorbers Operating at Visible Frequencies. IEEE Photonics Technol. Lett..

[18] Chen Z., Cai P., Wen Q., Chen H., Tang Y., Yi Z., Wei K., Li G., Tang B., Yi Y. (2023). Graphene Multi-Frequency Broadband and Ultra-Broadband Terahertz Absorber Based on Surface Plasmon Resonance. Electronics.

[19] Luo X., Tan R., Li Q., Chen J., Xie Y., Peng J., Zeng M., Jiang M., Wu C., He Y. (2023). High-sensitivity long-range surface plasmon resonance sensing assisted by gold nanoring cavity arrays and nanocavity coupling. Phys. Chem. Chem. Phys..

[20] Lai R., Shi P., Yi Z., Li H., Yi Y. (2023). Triple-Band Surface Plasmon Resonance Metamaterial Absorber Based on Open-Ended Prohibited Sign Type Monolayer Graphene. Micromachines.

[21] Tu M.-C., Cheema J.A., Yildiz U.H., Palaniappan A., Liedberg B. (2017). Vapor phase solvatochromic responses of polydiacetylene embedded matrix polymers. J. Mater. Chem. C.

[22] Tjandra A.D., Pham A.-H., Chandrawati R. (2022). Polydiacetylene-Based Sensors To Detect Volatile Organic Compounds. Chem. Mater..

[23] Finney T.J., Parikh S.J., Berman A., Sasaki D.Y., Kuhl T.L. (2021). Characterizing and Tuning the Properties of Polydiacetylene Films for Sensing Applications. Langmuir.

[24] Qian X., Städler B. (2019). Recent Developments in Polydiacetylene-Based Sensors. Chem. Mater..

[25] Mergu N., Kim H., Ryu J., Son Y.-A. (2020). A simple and fast responsive colorimetric moisture sensor based on symmetrical conjugated polymer. Sens. Actuators B Chem..

[26] Park D.-H., Heo J.-M., Jeong W., Yoo Y.H., Park B.J., Kim J.-M. (2018). Smartphone-Based VOC Sensor Using Colorimetric Polydiacetylenes. ACS Appl. Mater. Interfaces.

[27] Eaidkong T., Mungkarndee R., Phollookin C., Tumcharern G., Sukwattanasinitt M., Wacharasindhu S. (2012). Polydiacetylene paper-based colorimetric sensor array for vapor phase detection and identification of volatile organic compounds. J. Mater. Chem..

[28] Smith P.A. (2022). Intra-workday fluctuations of airborne contaminant concentration and the time-weighted average. J. Occup. Environ. Hyg..

[29] Wang S., Romanak K.A., Stubbings W.A., Arrandale V.H., Hendryx M., Diamond M.L., Salamova A., Venier M. (2019). Silicone wristbands integrate dermal and inhalation exposures to semi-volatile organic compounds (SVOCs). Environ. Int..

[30] Alabdulhadi A., Ramadan A., Devey P., Boggess M., Guest M. (2019). Inhalation exposure to volatile organic compounds in the printing industry. J. Air Waste Manag. Assoc..

[31] Vermeulen M., Klöpping-Ketelaars I.W.A.A., van den Berg R., Vaes W.H.J. (2008). Bioavailability and Kinetics of Sulforaphane in Humans after Consumption of Cooked versus Raw Broccoli. J. Agric. Food Chem..

[32] Röder-Stolinski C., Fischäder G., Oostingh G.J., Eder K., Duschl A., Lehmann I. (2008). Chlorobenzene induces the NF-kappa B and p38 MAP kinase pathways in lung epithelial cells. Inhal. Toxicol..

[33] Fischäder G., Röder-Stolinski C., Wichmann G., Nieber K., Lehmann I. (2008). Release of MCP-1 and IL-8 from lung epithelial cells exposed to volatile organic compounds. Toxicol. Vitr..

[34] Lehmann I., Röder-Stolinski C., Nieber K., Fischäder G. (2008). In vitro models for the assessment of inflammatory and immuno-modulatory effects of the volatile organic compound chlorobenzene. Exp. Toxicol. Pathol..

[35] Boyce B.F., Xiu Y., Li J., Xing L., Yao Z. (2015). NF-κB-Mediated Regulation of Osteoclastogenesis. Endocrinol. Metab..

[36] United States Department of Health and Human Services (2020). Toxicological Profile for Chlorobenzene.

[37] Pommier S., Llamas A.M., Lefebvre X. (2010). Analysis of the outcome of shredding pretreatment on the anaerobic biodegradability of paper and cardboard materials. Bioresour. Technol..

[38] Vinet A., Vézina M. (1989). Disability among female garment workers: A comparison with a national sample. Scand. J. Work. Environ. Health.

[39] Smith J.T., Schneider A.D., Katchko K.M., Yun C., Hsu E.L. (2017). Environmental Factors Impacting Bone-Relevant Chemokines. Front. Endocrinol..

[40] Arabi M., Alghamdi M., Kabel K., Labena A., Gado W.S., Mavani B., Scott A.J., Penlidis A., Yavuz M., Abdel-Rahman E. (2022). Detection of Volatile Organic Compounds by Using MEMS Sensors. Sensors.

[41] Vanduchova A., Anzenbacher P., Anzenbacherova E. (2018). Isothiocyanate from Broccoli, Sulforaphane, and Its Properties. J. Med. Food.

[42] Żebrowska A., Sadowska-Krępa E., Stanula A., Waśkiewicz Z., Łakomy O., Bezuglov E., Nikolaidis P.T., Rosemann T., Knechtle B. (2020). The effect of vitamin D supplementation on serum total 25(OH) levels and biochemical markers of skeletal muscles in runners. J. Int. Soc. Sports Nutr..

[43] Close G.L., Leckey J., Patterson M., Bradley W., Owens D.J., Fraser W.D., Morton J.P. (2013). The effects of vitamin D(3) supplementation on serum total 25[OH]D concentration and physical performance: A randomised dose-response study. Br. J. Sports Med..

[44] Ramasamy I. (2020). Vitamin D Metabolism and Guidelines for Vitamin D Supplementation. Clin. Biochem. Rev..

[45] Mangla B., Javed S., Sultan M.H., Kumar P., Kohli K., Najmi A., Alhazmi H.A., Al Bratty M., Ahsan W. (2021). Sulforaphane: A review of its therapeutic potentials, advances in its nanodelivery, recent patents, and clinical trials. Phytother. Res..

[46] Weiler J., Mohr M., Zänker K.S., Dittmar T. (2018). Matrix metalloproteinase-9 (MMP9) is involved in the TNF-α-induced fusion of human M13SV1-Cre breast epithelial cells and human MDA-MB-435-pFDR1 cancer cells. Cell Commun. Signal..

[47] Yamamoto H., Omelchenko I., Shi X., Nuttall A.L. (2009). The influence of NF-kappaB signal-transduction pathways on the murine inner ear by acoustic overstimulation. J. Neurosci. Res..

[48] Zafari V., Hashemzadeh S., Hosseinpour Feizi M., Pouladi N., Rostami Zadeh L., Sakhinia E. (2017). mRNA expression of nuclear factor of activated T-cells, cytoplasmic 2 (NFATc2) and peroxisome proliferator-activated receptor gamma (PPARG) transcription factors in colorectal carcinoma. Bosn. J. Basic Med. Sci..

