# Supplementary material for: Wearable Device for Cumulative Chlorobenzene Detection and Accessible Mitigation Strategies
Source: Sensors (Basel). 2023 Sep 15;23(18):7904. doi: 10.3390/s23187904 (PMC10536231; doi:10.3390/s23187904)
Supplement: Supplementary file 1 [file sensors-23-07904-s001.zip › sensors-2590423-supplementary.pdf]

Supplemental Information

**Supplemental Table S1:** Mouse Primer Sequences (RT-PCR) [46–48]		
Primer	Forward Sequence	Reverse Sequence
NF-κB	GAAATTCCTGATCCAGACAAAAAC	ATCACTTCAATGGCCTCTGTGTAG
Ctsk	AGCAGAACGGAGGCATTGACTC	CCCTCTGCATTTAGCTGCCTTTG
Mmp-9	TTCCAAACCTTTGAGGGCGA	CAAAGGCGTCGTCAATCACC
Ccl-2	GCTCAGCCAGATGCAATCAATG	GTGTCTGGGGAAAGCTAGGG
Nfatc	GGTGCCTTTTGCGAGCAGTATC	CGTATGGACCAGAATGTGACGG
Gapdh	ACTGAGCAAGAGAGGCCCTA	TATGGGGGTCTGGGATGGAA

Supplemental Table S2: RGB Color Values of PDA Arrays after Chlorobenzene Exposure, in described groups

	Ctrl			A			B			C		
DA 1: Red	72.6	68.4	69.2	90.4	92.0	91.1	108.7	107.7	109.6	112.6	116.6	112.3
DA 1: Green	110.3	112.0	113.1	123.6	124.8	123.0	130.1	130.1	127.0	143.7	141.2	145.2
DA 1: Blue	146.1	147.2	147.1	154.4	153.0	152.4	149.3	148.3	146.0	148.9	146.0	147.6
DA 2: Red	120.4	117.1	119.0	109.9	110.1	111.1	78.0	78.5	76.3	61.8	66.4	64.7
DA 2: Green	154.6	154.4	155.0	145.5	141.7	142.0	117.7	116.5	116.2	106.8	109.3	109.7
DA 2: Blue	187.1	183.8	188.8	168.8	168.0	168.5	155.2	158.1	157.5	152.6	151.0	152.4
DA 3: Red	101.5	99.2	96.6	162.8	165.7	165.0	201.6	201.6	203.9	203.6	201.3	204.0
DA 3: Green	149.0	148.7	151.1	138.7	134.1	138.7	108.6	108.1	109.9	84.3	82.7	80.1
DA 3: Blue	187.5	188.1	187.3	143.7	143.0	141.6	97.1	101.0	98.5	73.9	72.0	69.7
DA 4: Red	69.7	72.6	72.2	100.0	95.9	97.7	99.8	99.6	103.4	164.3	164.2	159.6
DA 4: Green	111.6	113.4	110.4	115.1	113.5	117.9	108.6	109.1	108.3	127.8	132.8	132.6
DA 4: Blue	149.6	150.8	149.5	134.1	133.2	134.6	114.1	113.7	110.1	128.8	124.2	127.0

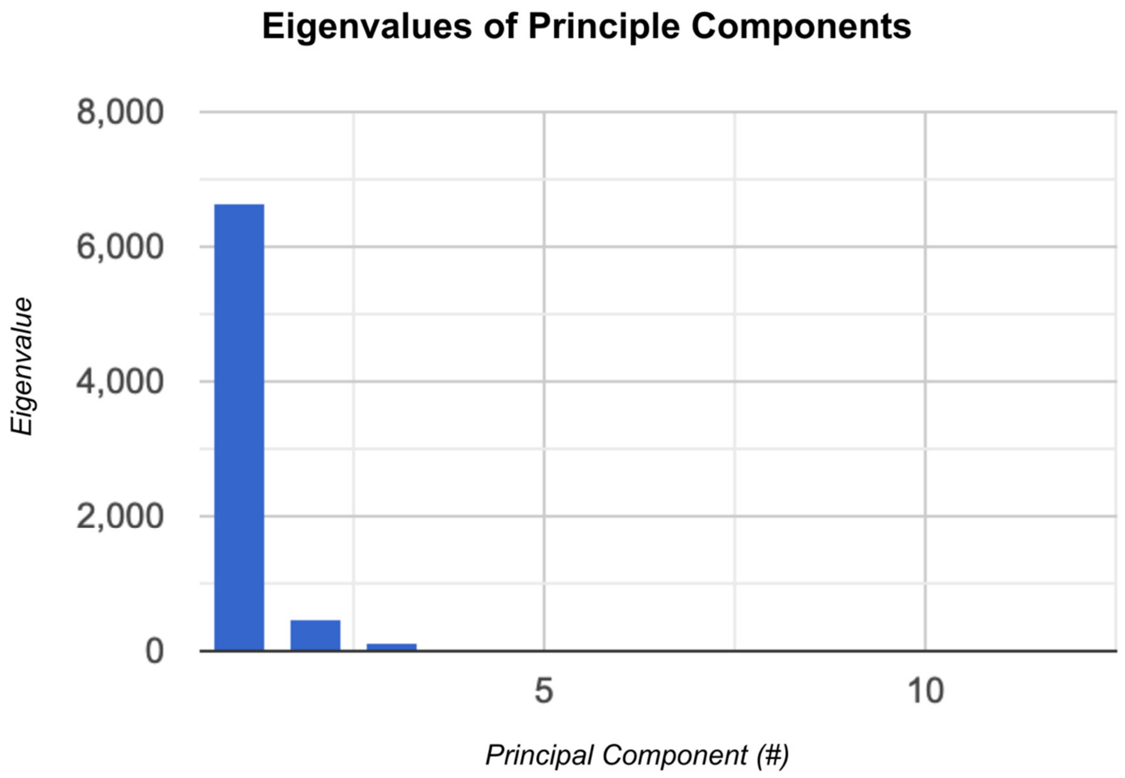

Supplemental Figure S1: Calculated Eigenvalues of each principal component in the covariance matrix. Eigenvalues signify the portion of the total variance that each Principal Component (each axis in Figure 3) captures.
